# Early evening light mitigates sleep compromising physiological and alerting responses to subsequent late evening light

**DOI:** 10.1038/s41598-019-52352-w

**Published:** 2019-11-05

**Authors:** Marije te Kulve, Luc J. M. Schlangen, Wouter D. van Marken Lichtenbelt

**Affiliations:** 1 Department of Human Biology & Movement Sciences, NUTRIM, Maastricht University, Maastricht, The Netherlands; 2 bba indoor environmental consultancy, The Hague, The Netherlands; 3 Intelligent Lighting Institute, Department of Human Technology Interaction, Eindhoven University of Technology, Eindhoven, The Netherlands; 4 Signify, Eindhoven, The Netherlands

**Keywords:** Biophysics, Physiology, Metabolism

## Abstract

The widespread use of electric light and electronic devices has resulted in an excessive exposure to light during the late-evening and at night. This late light exposure acutely suppresses melatonin and sleepiness and delays the circadian clock. Here we investigate whether the acute effects of late-evening light exposure on our physiology and sleepiness are reduced when this light exposure is preceded by early evening bright light. Twelve healthy young females were included in a randomised crossover study. All participants underwent three evening (18:30-00:30) sessions during which melatonin, subjective sleepiness, body temperature and skin blood flow were measured under different light conditions: (A) dim light, (B) dim light with a late-evening (22:30-23:30) light exposure of 750 lx, 4000 K, and (C) the same late-evening light exposure, but now preceded by early-evening bright light exposure (18.30-21.00; 1200 lx, 4000 K). Late-evening light exposure reduced melatonin levels and subjective sleepiness and resulted in larger skin temperature gradients as compared to dim. Interestingly, these effects were reduced when the late-evening light was preceded by an early evening 2.5-hour bright light exposure. Thus daytime and early-evening exposure to bright light can mitigate some of the sleep-disruptive consequences of light exposure in the later evening.

## Introduction

Light is an important zeitgeber (“time giver”) that adjusts the human internal circadian clock to a 24 hour period^1^. Depending on its timing, light exposure will differently affect circadian rhythms^2^. Light in the evening can delay the biological clock while morning light can result in a phase advance (e.g.^2,3^). The time of melatonin onset under dim light (DLMO) and the time of the minimum core body temperature (CBT) are important commonly used markers of the circadian clock^4^.Misalignment between the biological clock and behavioural rhythms not only induces sleep disturbances and daytime sleepiness, but can also result in desynchronization of internal rhythms (for example when comparing the central clock to peripheral clocks)^5^. There are indications that circadian misalignment has adverse cardio-metabolic consequences^6,7^ and that the circadian timing of food intake affects body weight^8^. Therefore, it is important to synchronize the central circadian clock with behavioural rhythms.

The human circadian system has evolved through entrainment by the natural light-dark cycle and seasonal influences^9^. However, currently people spend most of their times indoors (on average 87%)^10^. Daytime indoor light intensities are commonly much lower compared to outdoor light exposures, while evening light exposures (artificial light sources and light emitting screens) are relatively high and thereby can delay our circadian system^2,11^ and can acutely suppress melatonin levels and subjective sleepiness^12,13^. We hypothesize that the acute physiological and alerting effect of evening light exposure will be reduced by high daytime light intensities.

There are some studies that provide evidence for this hypothesis. The magnitude of circadian phase shifts induced by different combinations of evening and morning light exposure is found to be significantly smaller for blue-enriched morning light of 750 lx as compared to warm-white morning light of 40 lx^14^. Low daytime light levels for 3–7 days resulted in larger light induced melatonin suppression during the night as compared to higher daytime light levels^15–17^. Moreover, melatonin suppression by nocturnal light exposure is found to be lower when preceded by dim light adaptation instead of dark adaptation^18^. In accordance with these results, a study comparing indoor workers and outdoor workers revealed that higher 24-h light exposures were associated with less evening light induced melatonin suppression^19^. Finally, it was observed that the alerting effects of light (as measured by subjective sleepiness, performance tests and electroencephalogram) were greater and/or lasted longer when the prior light exposure was 1 lux compared to 90 lux^20^. Altogether, these studies show a consistent finding that daytime bright light reduces the sensitivity to light in the evening or night. Moreover, there are quite some studies that report daytime light exposure to be supportive for good sleep and wellbeing^21–25^. However, most people spend a large part of their days indoors in public settings (like workplaces or schools), usually under much lower light levels than outdoors, with little ability to control their lighting conditions. After a day at work or school, people are more likely to spend some time outdoors to commute or for leisure activities, enabling them to harvest some bright light exposure. Moreover, a (bright) light intervention is more easy to realize in a domestic setting (in the early evening at home,) than during daytime in a public setting. Therefore we explored to what extent early evening bright light can reduce the sleep disruptive effects of light exposure in the late(r) evening.

Night-time or evening bright light exposure does not only result in melatonin suppression and reduced sleepiness, it is also associated with a delay in the natural decline in CBT and higher distal proximal skin temperature gradient (DPG) (see^26^ for review). These effects of light are strongly mediated via the intrinsic photosensitive retinal ganglion cells (ipRGC’s), which contain the photopigment melanopsin with a peak sensitivity for wavelengths around 480 nm^27^. Indeed, short wavelength light exposure can suppress the increase in DPG (suppression of evening vasodilation) and distal skin temperatures^13,28^. Vasomotion can also be measured using skin blood flow (SBF) measurements. If evening light influences DPG, it is expected that it also affects SBF (see^29^ for a review on mechanisms of cutaneous vasomotion). Experimental studies have confirmed that SBF rises in the evening^30,31^, but to our knowledge no studies on the effects of evening light on SBF have been published so far. In summary, human body temperature (distribution) can be influenced by evening light. It is however unknown whether this influence of evening/nighttime light exposure can be reduced by exposure to high light intensities in the preceding daytime or early evening.

The objective of the current study is to test whether the physiological and alerting effects of a one-hour light exposure in the late evening depend on the light exposure in the preceding 4 hours. It is expected that evening light exposure reduces melatonin, SBF, DPG, distal skin temperatures and subjective sleepiness as compared to dim light. It is hypothesized that this reduction is less when the evening light exposure is preceded by a 2.5-hour bright light exposure in the early evening. To test this hypothesis, the current study evaluates thermo-physiological responses and subjective sleepiness during a one-hour evening light exposure (750 lx), with and without prior bright light exposure (1200 lx) in the early evening

## Results

### Melatonin

**Figure 1:**
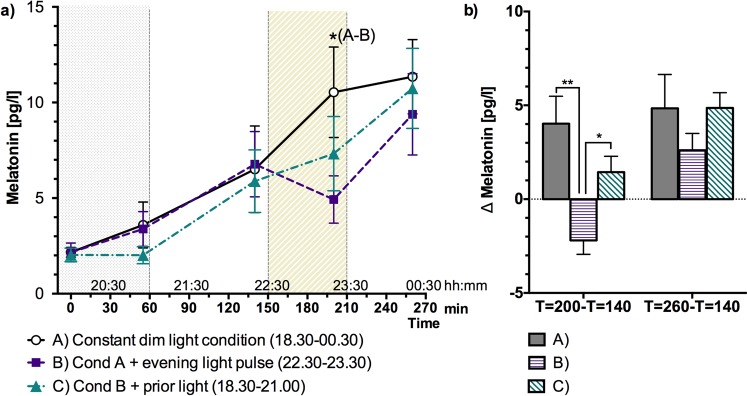
(**a**) Average melatonin concentration (mean ± SEM) as a function of time for all three sessions. The grey dotted area (T ≦ 60) indicates the last part of the 2.5 hr prior light exposure in session C and the diagonally hatched area indicates the timing of the 1 h light exposure period. (**b**) Change in melatonin concentration (mean ± SEM) from the 3rd sampling till the 4th (T = 200-T = 140) and from the 3rd till the 5th (T = 260-T = 140). Statistically significant differences between sessions are indicated: ^#^p < 0.10, *p < 0.05 and **p < 0.01.

Evening melatonin levels were significantly affected by light exposure. During one-hour light exposure (T = 200) (session B), mean melatonin concentration was lower than for the dim light exposure (session A) (melatonin suppression 34% Fig. 1a). Interestingly, melatonin concentration during the prior bright light session (C), was between the mean concentrations of session A and B, yet not significantly different from either (melatonin suppression 22%, Fig. 1a). The increase in melatonin concentration from T = 140 till T = 200 was largest when exposed to dim light (session A, Fig. 1b). Melatonin levels declined during the light exposure period when exposed to 750 lux light (session B, Fig. 1b). The change in melatonin concentration significantly differed between dim light and light (750 lux) exposure (A and B) and between light (750 lux) exposure with and without prior bright light exposure (B and C) (Fig. 1b). However, the change in melatonin concentration between the end of the experiment and just before light exposure (T = 260-T = 140) was not significantly different for the three evening light sessions.

The area under the curve (AUC) of the melatonin concentration before light exposure (i.e, between T = 0 and T = 140) was not significantly lower during the prior light exposure (C) as compared to the dim light sessions (A and B). This indicates that the prior light exposure by itself did not significantly suppress melatonin concentrations. During the light exposure (i.e, between T = 140 and T = 200) the AUC in session B tended to be lower as compared to the dim light session A (p = 0.052), for session C this tendency was not observed. Both the total AUC of the melatonin profile as well as the timepoint at which the melatonin profile exceeded 4 pg/l were not significantly different between the light sessions (p > 0.10 for all comparisons, see Table 1). For each session (A-C) the melatonin concentration profiles (pg/l) of individual participants are provided in the Supplementary Materials.

**Table 1:** The timepoints in sessions A, B and C at which the melatonin profile of an individual participant exceeded 4 pg/l, established using linear interpolation between two succeeding sample points of a participant.

Participant	Session A	Session B	Session C
1	0:20	0:20	0:20
2	23:44	22:00	23:29
3	23:23	0:09	0:00
4	20:12	20:20	21:12
5	21:27	0:20	0:15
6	20:59	21:07	23:24
7	23:59	0:20	0:20
8	20:11	21:21	21:03
9	20:08	20:09	21:22
10	22:36	21:01	20:00
11	22:34	23:44	20:30
12	21:33	21:24	22:26
Mean ± SD	22:05 ± 1:33	22:21 ± 1:41	22:31 ± 1:38

Under dim light (i.e., in session A) this time is equivalent to the DLMO^32^. SD is presented in hh:mm.

### Skin blood flow

**Figure 2:**
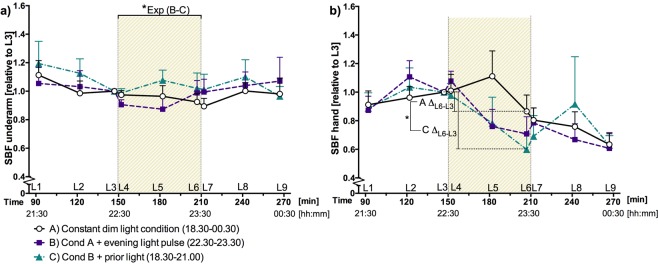
(**a**) SBF of the underarm, interval means + SEM per session over time. (**b**) SBF of the hand, interval means + SEM per session over time. SBF is presented relative to the 3rd LDF measurement interval (just before the light exposure period at T = 150). The diagonally hatched area indicates the light exposure period. Statistically significant differences between sessions are indicated: *p < 0.05.

Skin blood flow of the underarm did not differ much between the sessions, though during the prior light session (C) SBF was significantly higher during the 1 hour light exposure as compared to session B with preceding dim light (Fig. 2a). Skin blood flow of the hand did not differ significantly between sessions (Fig. 2b). The decline in SBF of the hand during the light exposure period (L6-L3) was larger for session C (prior light history) compared to the dim light exposure (session A) (Fig. 2b). The change in the SBF of the underarm was not different between the sessions.

### Body temperature, energy expenditure and heart rate

**Figure 3:**
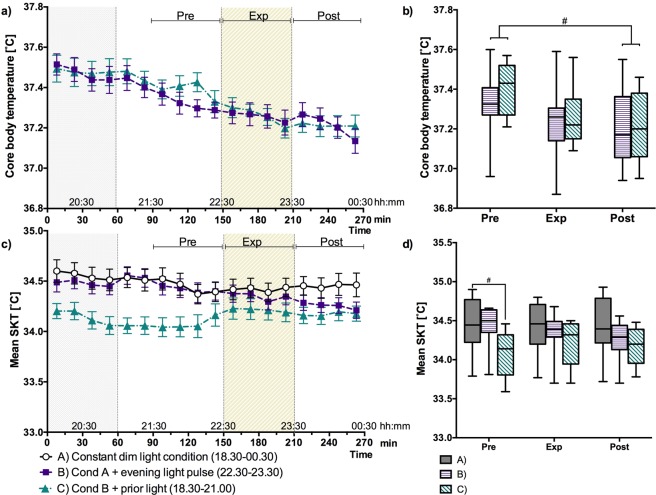
(**a**) Core body temperature 15-minute means ± SEM per session over time. (**b**) Boxplots of the 1-hour intervals of CBT per session. (**c**) Mean skin temperature 15-minute means ± SEM per session over time. (**d**) Boxplots of the 1 hour intervals of mean SKT per session. The grey dotted area (T < 60) indicates the prior light exposure and the diagonally hatched area indicates the timing of the light exposure period (**a**,**c**). Statistically significant differences between sessions and 1-hour means are indicated: ^#^p < 0.10, *p < 0.05 and **p < 0.01.

CBT declined during the evening (p < 0.10) (Fig. 3a,b). At each time point CBT was not significantly different between the two sessions (B and C). Also, the changes in CBT did not differ significantly between sessions. So, the effect of light exposure on CBT was not different when it was preceded by bright light. During the “pre” interval of session C, the mean SKT was lower as compared to the dim light session (A) (Fig. 3c,d). The 1 hour means of EE and HR were not significantly affected by the light exposures (all p > 0.10) (Table 2).

**Table 2:** Proximal SKT, Distal SKT, Energy expenditure (EE) and heartrate (HR), 1 hour mean ± SEM for each session during the “Pre”, “Exp” and “Post” intervals.

		Session A Mean ± SEM	Session B Mean ± SEM	Session C Mean ± SEM
Prox SKT [°C]	Pre	34.9 ± 0.06	34.9 ± 0.08	34.6 ± 0.10
	Exp	34.9 ± 0.08	34.8 ± 0.07	34.7 ± 0.11
	Post	35.0 ± 0.08	34.8 ± 0.09	34.7 ± 0.08
Distal SKT [°C]	Pre	33.1 ± 0.25	33.3 ± 0.27	32.8 ± 0.33
	Exp	33.1 ± 0.26	33.2 ± 0.23	33.0 ± 0.30
	Post	33.2 ± 0.26	32.7 ± 0.25	32.8 ± 0.25
EE [kj/min]	Pre	5.4 ± 0.14	5.1 ± 0.11	5.4 ± 0.10
	Exp	5.3 ± 0.13	5.2 ± 0.17	5.2 ± 0.14
	Post	5.3 ± 0.17	5.2 ± 0.13	5.2 ± 0.11
HR [bpm]	Pre	66 ± 3	67 ± 3	66 ± 3
	Exp	65 ± 3	66 ± 3	64 ± 3
	Post	67 ± 3	66 ± 3	64 ± 3

**Figure 4:**
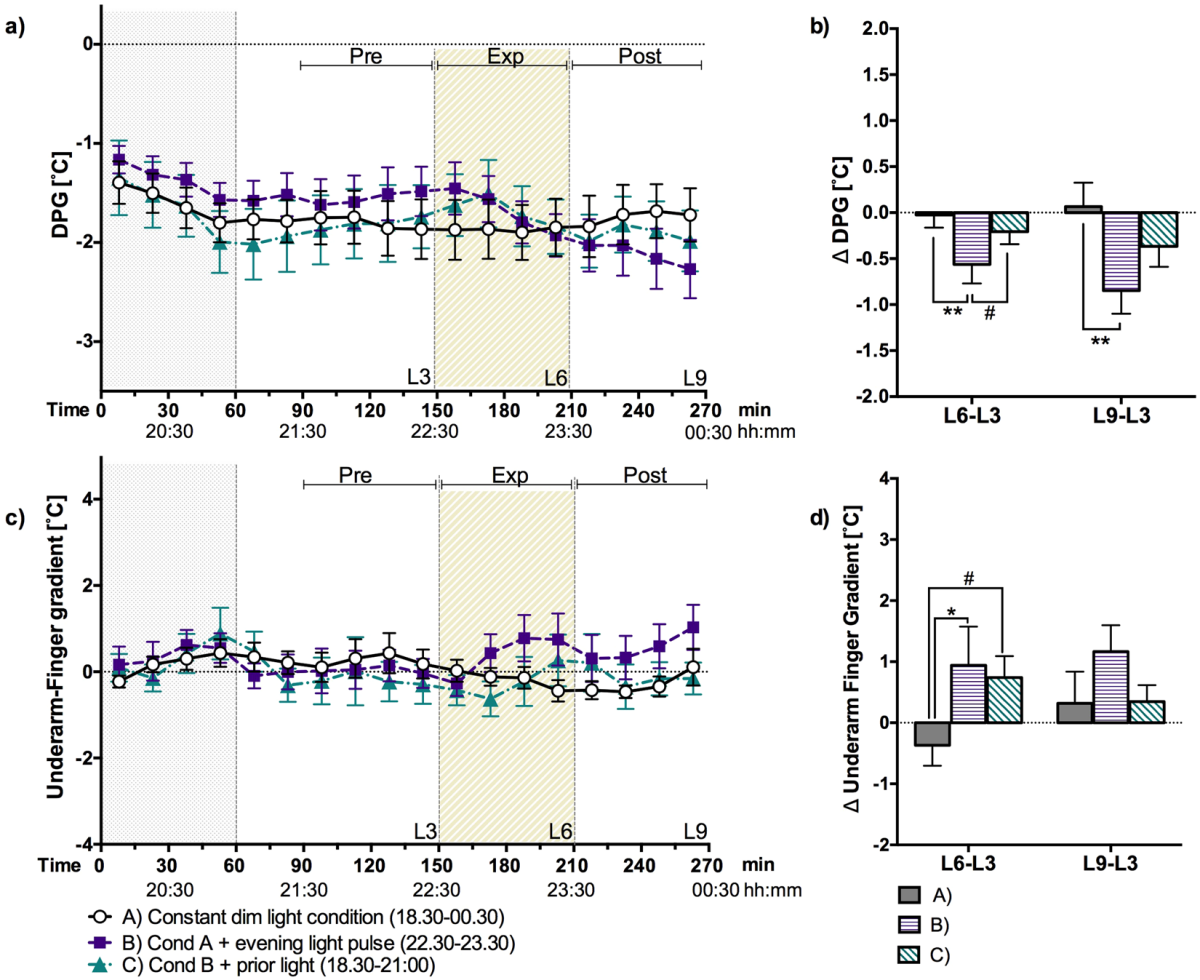
(**a**) Distal proximal skin temperature gradient (DPG) 15-minute means ± SEM per session over time. (**b**) The change in DPG during the exposure period (L6-L3) and till the end of the experiment (L9-L3). (**c**) Underarm-Finger gradient 15-minute means ± SEM per session over time. (**d**) The change in underarm-finger gradient during exposure period (L6-L3) and till the end of the experiment (L9-L3). The grey dotted area (T ≦ 60) indicates the prior light exposure and the diagonally hatched area indicates the timing of the light exposure period (**a**,**c**). Statistically significant differences between sessions and between 1-hour means are indicated: ^#^p < 0.10, *p < 0.05 and **p < 0.01.

The DPG and the underarm-finger gradient were not significantly affected by clock time (Fig. 4a,c). During the dim light session (A), the changes in both gradients was close to zero (Fig. 4b,d). During the evening light exposure (session B), the DPG declined, and the underarm finger gradient significantly increased (both indications for distal vasoconstriction) as compared to dim light (session A) (Fig. 4b,d). However, the decline in DPG during the light exposure period tended to be smaller after prior light exposure (session C compared B) (Fig. 4b). The increase of the underarm-finger gradient during the light exposure period after prior light exposure (session C), was in-between session A and B. The change during session C tended to be larger compared to dim light (A) but was not significantly different from session B. The decline of the DPG from the start of the light exposure period (T = 150) and the end of the experiment, was still larger for session B as compared session A (p < 0.01) (Fig. 4b). In conclusion, these results indicate that prior light exposure reduces distal vasoconstriction induced by subsequent evening light.

### Subjective sleepiness

**Figure 5:**
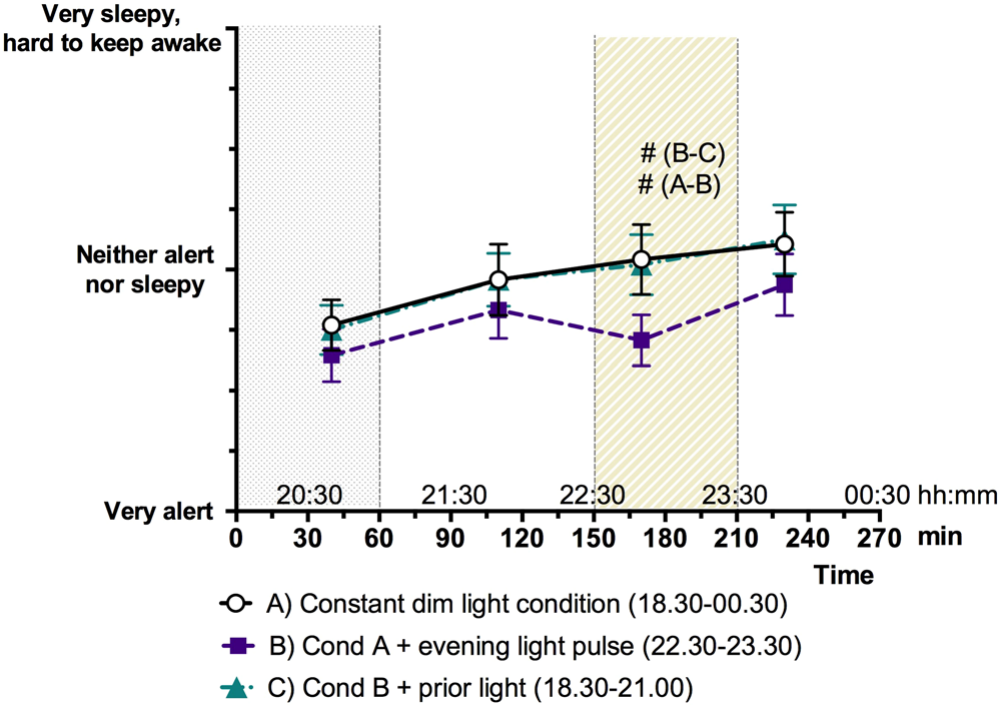
Subjective sleepiness mean ± SEM per session for each questionnaire. The grey dotted area (T ≦ 60) indicates the prior light exposure and the diagonally hatched area indicates the timing of the light exposure period (T = 150–210). Trends are indicated using ^#^p < 0.10.

Subjective sleepiness tended to be lower during one-hour light exposure (session B) as compared to dim light (session A) and to prior light (session C) (Fig. 5).

## Discussion

In the current study, we tested the effect of light exposure on sleepiness and thermophysiological parameters. Evening light (compared to dim light) was found to reduce melatonin levels, decrease subjective sleepiness (trend), reduce the DPG and increase the underarm-finger SKT gradient (indicative of more distal vasoconstriction). Interestingly, when preceded by bright light in the early evening, the effects of one-hour light exposure (in the late evening) on thermophysiology and on subjective sleepiness were reduced.

Evening light exposure (bright light or light rich of short wavelengths of ~480 nm) is known to suppress melatonin production (e.g.^33,34^), reduce the decline in CBT (e.g.^35–37^) and reduce sleepiness^38^. Our study results are in line with the literature and confirm the effect of evening light on thermophysiology. The skin temperature gradients were larger during light exposure, indicative of distal vasoconstriction of blood vessels. In the evening, under a low light intensity (<8 lux), distal skin temperatures naturally rise, thereby increasing the DPG (to become less negative) and heat loss^39^. In the current experiment, bright light exposure suppressed this natural increase in DPG. Altogether our study results show that the 750 lux light exposure was effective in suppressing melatonin production, reducing subjective sleepiness and affecting body temperatures in women.

We hypothesized that larger skin temperature gradients would go along with a lower SBF. However, in our results, we did not observe a statistically significant difference in SBF between the dim light session (A) and the 750 lx light exposure session (B). The change in vasoconstriction can have been too small to observe in the SBF when using LDF or can have been not perceptible at this particular location. In addition, it must be noted that the SBF data are presented as a relative value using the interval just before the 1-hour light exposure (L3) as a reference. However, Fig. 2a shows that the (relative) SBF data before L3 do differ between the sessions with (session C) and without (session A and B) prior light exposure. This indicates that the L3 reference could already include effects of the prior light, this then would also affect the relative values during the subsequent light exposure. Therefore, prior light exposure effects within the SBF reference can have contributed to the significant difference between session B and C in Fig. 2a. This effect can also obscure a direct comparison between sessions A and B. For future studies it is recommended to measure the reference value before any (light) intervention.

A bright light history in the early evening can decrease the (sleep disruptive) effects of light exposure in the subsequent late evening. This is in accordance with previous studies^15–17,20^, where prior (daytime) light exposure has been shown to reduce responses to late evening light, as already mentioned in the introduction. However, in the current study we show that a single 2.5-hour bright light exposure in the early evening (ending 1.5 hours before the one-hour lasting late-evening light exposure) already changes the response to this late evening light, with the prior light disempowering the subsequent light exposure. We also showed that prior light exposure influenced the evening modulation of skin temperature gradients. With prior light (session C), the skin temperatures gradients (absolute value of both the DPG and the underarm-finger gradient) increased less during the late evening light exposure as compared to without prior light (session B).

The prior-light-dependent thermophysiological and sleepiness responses to late evening light can, at least partly, be attributed to (light-driven) changes in melatonin concentration, since melatonin is associated with higher distal skin temperatures, smaller DPG, lower CBT and higher sleepiness^40,41^. The intrinsic photosensitive retinal ganglion cells (ipRGCs) are an important mediating factor in these melatonin responses. They directly project to the SCN, which coordinates the circadian rhythm of a wide range of physiological processes, including melatonin production^42,43^. The intrinsic light sensitivity of ipRGCs reduces in a constant bright background^44^. Similarly, a brighter prior light history could also reduce the intrinsic light sensitivity of the ipRGCs, thus explaining the drop in melatonin suppression when the late evening light is preceded by early evening bright light instead of dim light. Alternatively, an increase in daytime light exposure can increase nocturnal melatonin secretion^24^, and this could (at least partly) explain why, on the later evening, the melatonin levels in session C are higher than in session B (see Fig. 3a). In the early evening, during the prior light exposure, the average melatonin concentrations in session (C) were similar as compared to the dim light session (without prior light). Moreover, the early evening light as used in the current study did not result in a significant delay of the time where the average melatonin concentration in Fig. 3a passed the >4 pg/l threshold: for session C this time was virtually identical to the DLMO of session A. However, on an individual level the >4 pg/l time point did differ between the three light sessions (see Table 1). This indicates that the response of individual participants to evening light exposure largely differs between individuals, as also reported in a recent paper^45^. However, the group mean time point of the >4 pg/l threshold was not different between the sessions, see Table 1. Finally, we note that it would be interesting to investigate how the melatonin profile is affected when the prior light exposure is added to the dim light session without the 1 h late evening light exposure. More research is needed to establish the role of melatonin in the reduced thermophysiological and sleepiness response to late evening light after prior light exposure in the early evening.

The current experiment showed that late evening light acutely suppressed melatonin and increased skin temperature gradients. One hour post light exposure, at the end of the experiment/session (i.e, after having spent the final hour in dim light), the evening melatonin levels of the different light sessions converged. However, up to 1 hour after the late-evening-light exposure, the skin temperature gradients were larger in the session without prior light exposure. A larger skin temperature gradient can make falling asleep more difficult^46^. Therefore, it is important to further study the ability of prior light exposure (either during the early evening or during daytime) to reduce the (thermo) physiological effects of subsequent evening light exposure. In such further studies also the role of the spectral irradiance changes during natural twilight merits to be considered. Under natural conditions, the spectral changes during twilight are required for appropriate circadian entrainment (in mice^47^), and twilight transitions could also affect responses to evening/nighttime light exposures. Future studies on the effect of light history on evening light responses also need to consider gender, as previous studies demonstrated that women are less sensitive to the effect of evening light compared to male^48,49^. Moreover, to measure statistically significant differences in SBF may require a larger sample size than the one used in the present study.

In conclusion, a 2.5-hour bright light exposure during the 4-hours preceding a one-hour light exposure in the late evening can strongly reduce the influence of the late evening light exposure on body temperatures, melatonin and subjective sleepiness, thus mitigating the sleep disruptive effects of (late) evening light exposure. This study was the first to show that the effect of late evening light on thermophysiology, i.e. body temperature distribution, is affected by a few hours light exposure in the preceding early evening. These results imply that the adverse effects of late evening light exposure, which are a common phenomenon, can be mitigated by exposure to bright light during the preceding daytime or early part of the evening.

The phase delaying (and circadian rhythm disrupting) effects of late evening light exposure are smaller when this light exposure (i) is less bright^50^, (ii) has a shorter duration^51^, (iii) is with a longer wavelength^33,52^, or is blue-depleted^53^, (iv) is preceded by several days with higher daytime light exposures, and (v) occurs more early in the evening^17,54^. Further studies are required to test if a single prior bright light exposure in the early evening, or late afternoon, can be another strategy to reduce the disruptive phase delaying effects of subsequent late evening light exposures. The current study clearly indicates that seeking brighter light exposures (or being outdoors) during the late afternoon and/or early evening is a useful strategy to make the ubiquitous late evening light exposure within our society less sleep disruptive, especially when these late light exposures can’t be dimmed or blue-depleted.

## Methods

A randomised crossover study design was used to compare three sessions that differed in light exposure. Prior to the start of the study procedures, participants provided written informed consent to participate in the study. All procedures were conducted in accordance with the declaration of Helsinki. The Medical Ethical Committee of Maastricht University Medical Centre + approved the study protocol.

### Participants

Twelve healthy females participated in this study Inclusion criteria were: generally healthy, no medication use except from oral contraceptives (mandatory), BMI between 18–25 kg/m^2^, age 18–30 years old and a normal chronotype (reported bedtimes^55^ on weekdays between: 23:00 ± 00:30 till 7:00 ± 0:30 min and in weekends between: 23:30 ± 01:00 till 07:30 ± 01:00 min,). Reported bedtimes were not further verified, and need not represent the actual sleep (onset/offset) times. The inclusion and exclusion criteria were checked prior to inclusion of the study using a medical questionnaire and a chronotype questionnaire^55^. The subject characteristics are given in Table 3. All participants confirmed that they did not suffer from colour blindness, however this was not further verified with a test.

**Table 3:** Subject characteristics (N = 12).

	Mean ± SD	
Age	21.4 ± 2.14	years
Height	1.73 ± 0.04	meters
Weight	65.2 ± 6.41	kg
BMI	21.7 ± 1.93	kg/m^2^

### Procedure

**Figure 6:**
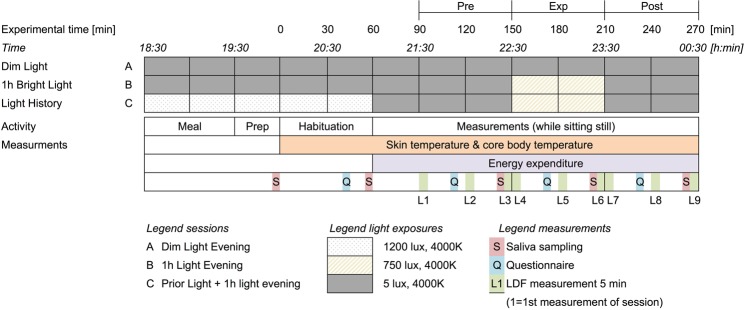
Scheme of the 3 light sessions and measurements during the experiments. The 1-hour intervals “Pre”, “Exp” (exposure period) and “Post” are indicated.

The experiments took place in the respiratory chambers of the Medical Research Unit Maastricht (MRUM) in which light and temperature can be controlled accurately. All participants underwent three evening sessions that differed in light exposure. Each evening sessions started at 18:30 h and lasted till 00:30 h at night (Fig. 6). During session A, participants were continuously exposed to dim light (5 lx, 4000 K). Session B was identical to session A, apart from an additional light exposure (750 lx, 4000 K) between 22:30 h and 23:30 h. Session C started with a bright light exposure (1200 lx, 4000 K) until 21:00 h, followed by dim light between 21:00 and 22:30, Between 22:30 and 23:30 another light exposure was given (750 lx, 4000 K) followed by dim light exposure until 00:30. The order of the sessions was randomised among participants. There was at least one week between two sessions and a maximum of four weeks between the first and the last session.

During the sessions, the participants arrived at 18:30 h at the laboratory. On the day of the experiment they refrained from sports, caffeine and alcohol and were not allowed to eat after 13:00 h, and had to spend the afternoon at the university. After arrival at the laboratory of the university, a standardized meal was provided. The caloric content of the meal was calculated for 6 hours using the revised version of the Harris and Benedict equation^56^. After the meal the participants were only allowed to drink water. Room temperature was set at the calculated midpoint of the thermo neutral zone (TNZ) of each participant using the model reported in^57^. The model takes into account gender, age, length, weight, activity and clothing. The midpoint of the TNZ was chosen, because of the large capacity of the body to increase or reduce heat loss by skin blood flow. Skin temperature sensors were attached to the body. From then on, the participants were sitting in a chair with a high backrest and foot support and the experiment started (T = 0). After one-hour, the probes of the Laser Doppler flowmetry (LDF) were attached to the hand and the underarm. The door of the respiration chamber was then closed from T = 60 till T = 270 so energy expenditure could be measured. During the experiments the participants were allowed to read and to listen to the radio. Instructions were given via an intercom.

### Measurements

#### Skin blood flow

Skin blood flow (SBF) was measured using laser Doppler flowmetry (LDF). Because this measure is highly sensitive to movements, 5 minutes periods for measuring SBF were included in the protocol (Fig. 6). During each period, the participant put her arm in an armrest to avoid movement of the arm. In total, there were 9 periods (L1–L9) (Fig. 6). The first started at T = 90. During the light switches, there were two immediately consecutive measurement periods to measure SBF just before and just after the change of light exposure. Since this measurement technique does not provide an absolute measurement of skin blood flow, the data was normalized to the last measurement before the light exposure period (L3). There were three sessions during which there was a failure of the LDF measurement of the hand and one of the underarm and could therefore not be included in the data analyses.

#### Thermophysiological parameters

Skin temperatures were measured continuously during the experiment using iButton dataloggers (iButton, DS1922L, Maxim) (Fig. 6). The iButtons were attached to 26 sides of the body (as reported in^58^). The distal skin temperature was calculated as the mean skin temperature of the hand and foot. For the proximal skin temperature, the mean skin temperature of the lower back, shoulder, abdomen and chest was used. The DPG is the distal skin temperature minus the proximal skin temperature. CBT was measured during the session B and session C, using a telemetric pill (VitalSense® medical grade capsules, Equivital^TM^, Hidalgo Limited). The pill was ingested just after arrival at the laboratory. There was one session of one participant during which the CBT pill did not record reliable data and was therefore excluded from the data analyses. Heartrate was measured using the Sensory Electronics Module of the Equivital EQ02 Life Monitor system (Equivital^TM^, Hidalgo Limited). Human energy expenditure was measured using indirect calorimetry starting at T = 60. Oxygen consumption and carbon dioxide production were measured with an automated respiratory gas analyser (Omnical Indirect Calorimeter, Maastricht Instruments). Energy expenditure was than calculated using the Weir’s method^59^. All thermophysiological parameters were measured with 1-minute intervals.

#### Melatonin

Saliva samples were collected 5 times during each session (T = 0, T = 55, T = 140, T = 200 and T = 260, see Fig. 6). Saliva was collected using a cotton ball which was put in the mouth for 1–3 minutes until it was soaked with saliva. The saliva tubes were stored in a freezer at −20 °C. Melatonin concentration was determined using double-antibody radio immunoassay based on the Kennaway G280 anti-melatonin antibody (Bühlmann direct Saliva Melatonin Radio Immunoassay (RIA) test kit).

#### Subjective sleepiness

Subjective sleepiness was evaluated using a questionnaire including the Karolinska sleepiness scale (KSS) ranging from 1 “Very alert” till 9 “Very sleepy and hard to keep awake”^60^. The questionnaire was filled out 4 times during the experiment (as indicated with “Q” in Fig. 6).

#### Light conditions

The lighting system consisted of eight LED wall washer fixtures (Philips SkyRibbon IntelliHue Wall Washing Powercore). The wall washing fixtures were mounted in the ceiling and provided only indirect light, they were illuminating the two walls on the long side of the windowless room (2.0 × 2.8 meters, height 2.1 m). All walls were painted white as to reflect the light of the wall washers into the room. The participants could not look directly into the fixtures.

The spectrum and the illuminance of the three different light condtions were measured at the height of the eye in the most likely direction of gaze (AsenseTEK, Lighting Passport, Allied Scientific Pro). The characteristics of the different light exposures are given in Table 4).

**Table 4:** Characteristics of the three different light conditions.

		Dim light	1 h light exposure (at 22:30)	Prior light exposure (18:30-21:00)
	Illuminance (lx)	5.0	777	1227
	CCT [K]	3888	3888	3934
Lucas *et al*., 2014	“Cyanopic-lx”	2.4	376	608
	“Melanopic-lx”	3.8	596	953
	“Rhodopic-lx”	4.2	651	1036
	“Chloropic-lx”	4.6	708	1122
	“Erythropic-lx”	4.9	764	1205
CIE S 026/E:2018^a^	S-cone-opic EDI (lx)	2.5	385	623
	M-cone-opic EDI (lx)	4.5	692	1096
	L-cone-opic EDI (lx)	5.0	779	1228
	Rhodopic EDI (lx)	3.8	590	939
	Melanopic EDI (lx)	3.5	540	863

Photopic illuminance (lx), correlated colour temperature (CCT) and the corresponding human retinal photoreceptor weighted “α-opic (equivalent) illuminances” and α-opic equivalent daylight (D65) illuminances (α-opic EDI) calculated according to Lucas *et al*.^61^ and CIE S 026/E:2018^62^ respectively.

^a^each α-opic EDI value gives the amount of lx of standard daylight D65 (phase of daylight with a CCT of approximately 6500 K) which results in an identical α-opic irradiance (i.e., the effective photobiological radiance with the spectral irradiance spectrally weighted with the α-opic action spectrum) as the test light condition, see international standard CIE S 026/E:2018^62^.

### Indoor temperature

The indoor temperature and relative humidity were measured using hygrobuttons (iButton, DS1923, Maxim). The hygrobuttons were fixed to a line at a height of 0.1 m, 0.6 m and 1.1 m at a horizontal distance of about 0.5 m from the participant. The mean (±SEM) ambient temperature of the sessions was 27.6 ± 0.02 °C (Table 5). For each interval (“Pre”, “Exp” and “Post’, see Fig. 6), a mixed model repeated over session was used to test whether there were significant (unintended) differences in ambient temperature between sessions. Though the results show only very small differences between sessions (all <0.40 °C), they were significant in some comparisons. Therefore, the ambient temperature was included as covariate in the skin temperatures analyses. Room temperatures were generally well fixed. However, due to large fluctuation of the indoor temperature (>1.0 °C between sessions of one participant), the data of the skin temperature and skin blood flow of four experimental sessions of three participants were excluded from the analyses.

**Table 5:** Ambient temperature during the experiments.

Session	Pre (T = 90-150)		Exp (T = 150–210)		Post (T = 210–270)	
	Mean ± SEM	Statistics	Mean ± SEM	Statistics	Mean ± SEM	Statistics
A	27.74 ± 0.08	A-B NS B > C Δ = 0.35 °C** A > C Δ = 0.39 °C**	27.76 ± 0.08	A-B NS B-C NS A > C Δ = 0.17 °C*	27.78 ± 0.07	A > B Δ = 0.33 °C** B-C NS A > C Δ = 0.28 °C**
B	27.68 ± 0.06		27.68 ± 0.05		27.43 ± 0.05	
C	27.29 ± 0.13		27.53 ± 0.09		27.43 ± 0.06	

Ambient temperature during the experiments for the 1 hour intervals as indicated in Fig. 6 (mean ± SEM in °C of all participants). Statistically significant differences between sessions are indicated: *p < 0.05, **p < 0.01 and NS: not significant.

### Data analyses

For the statistical analyses, comparisons between the following sessions were made:

- Session A and B to test the effect of one-hour evening light exposure compared to dim light exposure.
- Session B and C to test whether the effect of one-hour evening light exposure was affected by prior bright light exposure.
- Session A and C to test whether the effect of one-hour evening light exposure after prior bright light exposure was different from dim light exposure during the light exposure period.

The melatonin concentration of each saliva sample (5 per session) were analysed. The area under the curve (AUC) was calculated using the linear trapezoidal method. The % melatonin suppression was calculated from the AUC of the melatonin concentration during the 1 h evening light time slot, using the dim light session as the reference condition. The timepoint (in minutes) within each session at which the melatonin profile of each participant exceeded 4 pg/l was determined using linear interpolation. In the dim light session A this timepoint represents the DLMO and defined as the first time point that the melatonin concentration was >4 pg/l, using linear interpolation between two succeeding sample points^32^. Subjective sleepiness of each questionnaire was analysed for each timepoint. Five-minute SBF means (normalized values) of the 9 LDF measurement periods were used to calculate the 1 h means of SBF (intervals Pre, Exp and Post, see Fig. 6). For skin temperatures, CBT, SBF, EE and HR the 1-hour means were calculated. For all parameters, a linear mixed model repeated for all light sessions was applied to test for significant differences between sessions at each interval or time point. For the skin temperatures, the measured ambient temperature was included as a covariate to compensate for small fluctuations in ambient temperature. The effect of clock time during the experiment was tested using a linear mixed model repeated over time and session for the 1 hour means (“Pre”, “Exp” and “Post”). The change of the thermophysiological parameters was calculated using the last 5 minutes of each interval (L3, L6 and L9, Fig. 6) and analysed using mixed models. For the skin temperatures, the change in ambient temperature was included in the model as a covariate. The change in melatonin between the 3^rd^, 4^th^ and 5^th^ sampling was also calculated and analysed. All statistical analyses were performed using IBM SPSS Statistics 23. P-values < 0.05 were considered as statistically significant and p-values < 0.10 are reported as trends.

## Supplementary information

Supplementary Figure 1.
