# Supplementary material for: Early evening light mitigates sleep compromising physiological and alerting responses to subsequent late evening light
Source: Sci Rep. 2019 Nov 5;9:16064. doi: 10.1038/s41598-019-52352-w (PMC6831674; doi:10.1038/s41598-019-52352-w)
Supplement: Supplementary file 1 — Supplementary Figure 1. [file 41598_2019_52352_MOESM1_ESM.pdf]

Early evening light mitigates sleep compromising physiological and alerting responses to subsequent late evening light

Marije te Kulve[1,2], Luc J.M. Schlangen[3], Wouter D. van Marken Lichtenbelt[1]
1 Department of Human Biology & Movement Sciences, NUTRIM, Maastricht University, The Netherlands.
2 bba indoor environmental consultancy, The Hague, The Netherlands
3 Signify, Eindhoven, The Netherlands

Supplementary Figures

(A)

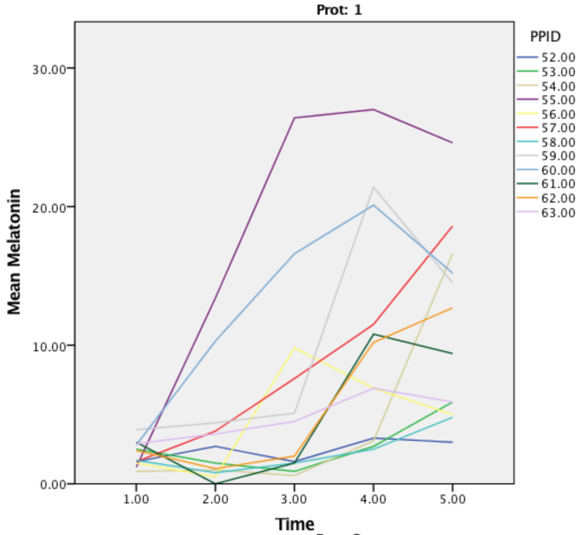

(B)

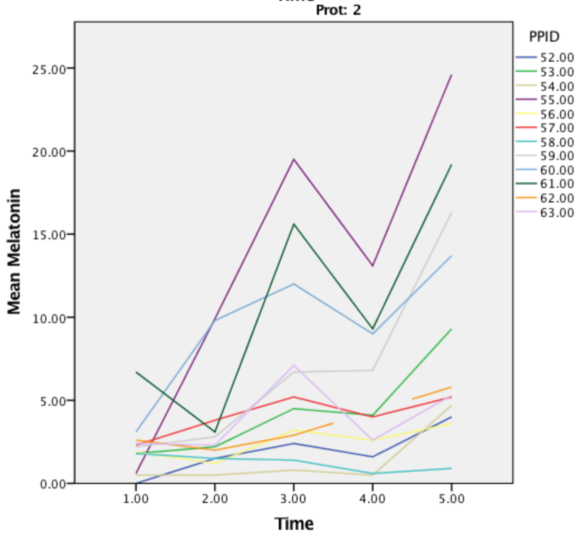

(C)

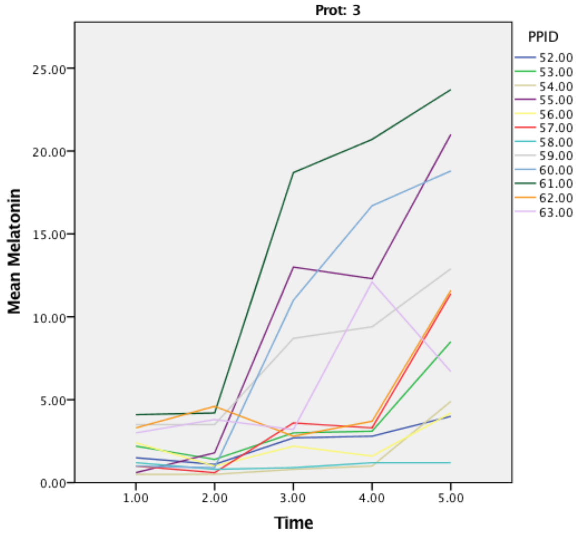

Supplementary Figure 1. Individual time-course of the melatonin concentration (pg/l) per session. (A) Melatonin concentration during dim light session. (B) Melatonin concentration during the 1 hour light session. (C) Melatonin concentration during the prior light exposure session. Each coloured line represents a participant.
